# Changes in the Golgi Apparatus of Neocortical and Hippocampal Neurons in the Hibernating Hamster

**DOI:** 10.3389/fnana.2015.00157

**Published:** 2015-12-15

**Authors:** Alejandro Antón-Fernández, Gonzalo León-Espinosa, Javier DeFelipe, Alberto Muñoz

**Affiliations:** ^1^Departamento de Neurobiología Funcional y de Sistemas, Instituto Cajal, CSICMadrid, Spain; ^2^Laboratorio Cajal de Circuitos Corticales, Centro de Tecnología Biomédica, Universidad Politécnica de MadridMadrid, Spain; ^3^Facultad de Farmacia, Universidad San Pablo CEUMadrid, Spain; ^4^Centro de Investigación Biomédica en Red de Enfermedades NeurodegenerativasMadrid, Spain; ^5^Departamento de Biología Celular, Facultad de Biología, Universidad ComplutenseMadrid, Spain

**Keywords:** hibernation, GM130, Golgin84, MG160, pyramidal neuron

## Abstract

Hibernating animals have been used as models to study several aspects of the plastic changes that occur in the metabolism and physiology of neurons. These models are also of interest in the study of Alzheimer's disease because the microtubule-associated protein tau is hyperphosphorylated during the hibernation state known as torpor, similar to the pretangle stage of Alzheimer's disease. Hibernating animals undergo torpor periods with drops in body temperature and metabolic rate, and a virtual cessation of neural activity. These processes are accompanied by morphological and neurochemical changes in neurons, which reverse a few hours after coming out of the torpor state. Since tau has been implicated in the structural regulation of the neuronal Golgi apparatus (GA) we have used Western Blot and immunocytochemistry to analyze whether the GA is modified in cortical neurons of the Syrian hamster at different hibernation stages. The results show that, during the hibernation cycle, the GA undergo important structural changes along with differential modifications in expression levels and distribution patterns of Golgi structural proteins. These changes were accompanied by significant transitory reductions in the volume and surface area of the GA elements during torpor and arousal stages as compared with euthermic animals.

## Introduction

The Golgi complex is a cellular organelle involved in the processing, modification, transport and targeting of cellular proteins. It is composed of stacks comprising closely apposed flattened cisternae and vesicles usually localized in the juxtanuclear area (Képès et al., 2005; Egea et al., 2006; Yadav and Linstedt, 2011) and held in position due to microtubule (MT)-dependent mechanisms. In mammalian cells, these stacks are laterally linked to form a membrane network, termed the Golgi ribbon, that allows an increase in the efficiency of glycosylation by creating enzymatic subcompartments in the order required for processing (Storrie et al., 1998; Storrie and Yang, 1998). Despite the degree of organization of the Golgi apparatus (GA), it has been shown that this organelle is highly dynamic and, under a variety of physiological processes, such as mitotic cell division (Levine et al., 1995), and pathological conditions, it can combine enormous rates of membrane flux with the ability to rapidly change its shape, redistribute different Golgi resident proteins (Glick, 2002) and even to disassemble and reassemble (Fan et al., 2008).

Hibernation is a physiological condition that allows hibernating mammalian species to survive in cold climates during times of reduced food availability. Hibernating animals have been used as models to study several aspects of plastic changes that occur in the metabolism and physiology of neurons in the normal brain under these conditions. In small mammals, hibernation is characterized by periods of reduced body temperature and metabolic rate, called torpor, that can last several days, interspersed with short arousal periods of activity and normothermia (Geiser, 2004, 2013; Geiser and Martin, 2013). Hibernation results in a decrease in metabolic rate (Zhou et al., 2001) and requires drastic physiological changes to maintain the energy savings (Geiser, 2004, 2013; Geiser and Martin, 2013). During torpor bouts, transcription and protein synthesis are severely depressed to keep energetically demanding cellular processes like transcription and translation to a minimum, with such processes recovering fully during the interbout arousal (Zhegunov, 1988; Rolfe and Brown, 1997; van Breukelen and Martin, 2001). With regard to the reduced protein synthesis, previous electron microscopy observations in taste bud cells (Popov et al., 1999) and in CA3 pyramidal neurons (Bocharova et al., 2011) revealed a transitory reduction in the number of polyribosomes and in rough endoplasmic reticulum profiles during torpor stages. Regarding the GA, a transitory fragmentation or disassembly along with a loss of flattened cisternae during torpor have been reported in studies using conventional electron microscopy (Popov et al., 1999; Bocharova et al., 2011). The structural maintenance of the Golgi ribbon has been shown to depend on protein complexes made by the 65kD Golgi reassembly stacking protein (GRASP65) and the 130 kD *cis*-Golgi matrix protein (GM130), both of which are mainly localized in the *cis*-Golgi compartment and are responsible for tethering lateral cisternae together, thereby promoting their fusion (Nakamura et al., 1995; Barr et al., 1997, 1998; Puthenveedu et al., 2006). The architecture of the GA has also been shown to depend on Golgin84, a rab protein present throughout the Golgi stacks with an increasing gradient toward the *trans*-side (Diao et al., 2003; Satoh et al., 2003; Sohda et al., 2010). Interference with Golgin84 expression has been shown to disrupt the Golgi ribbon and induce the appearance of Golgi fragments dispersed throughout the cell (Jiang et al., 2014). The distribution of these proteins in the neuronal GA of cortical neurons in hibernating animals is not known, and it is also not clear whether their expression and/or distribution change during the different phases of the hibernation cycle. In the present study we have used Western blot, immunocytochemistry and confocal microscopy to analyze the expression levels and distribution patterns of GM130 and Golgin84 in the Syrian hamster *Mesocricetus auratus* in torpor, arousal and euthermic states. Similarly we have analyzed the expression of MG160, a 160 kDa membrane sialoglycoprotein residing in the medial cisternae of the GA that is involved in the traffic, processing and probably in the regulation of endogenous or autocrine FGFs and that has been suggested to play important roles in the biogenesis and function of the GA (Gonatas et al., 1995, 1998a). The results indicate that the GA undergoes a profound and reversible morphological and neurochemical reorganization during the hibernation cycle that likely affects the ability to process and sort proteins.

In addition, mammalian hibernation has been proposed as a model to study certain physiological aspects of microtubule-associated protein tau phosphorylation *in vivo*. Indeed, this model may be useful to study the neuroprotection mechanisms in the first stages of neurodegenerative disorders such as Alzheimer's disease (AD), since neurons from hibernating animals show a transitory PHF (Paired Helical Filaments)-like tau phosphorylation with some similarities to that of patients at early stages of the disease (Zhou et al., 2001; Arendt et al., 2003; Avila et al., 2004; Härtig et al., 2005, 2007; Su et al., 2008; Stieler et al., 2011; León-Espinosa et al., 2013). Since tau has been localized in association with Golgi membranes, where it could serve as a link between these structures and microtubules (Farah et al., 2006), and the GA undergo widespread structural alterations in neurons of patients and animal models of AD (Gonatas et al., 1998b; Liazoghli et al., 2005; Hu et al., 2007), in the present study we analyze whether the alterations of the GA in cortical neurons of hibernating hamsters during torpor might be correlated with the accumulation of hyperphosphorylated tau.

## Materials and methods

All experimental procedures were carried out at the animal facility of the San Pablo CEU University of Madrid (SVA-CEU.USP, registration number ES 28022 0000015) and were approved by the institutional Animal Experiment Ethics Committee. A total of 21 male 4-month-old Syrian hamsters (*Mesocricetus auratus*) were purchased from Janvier Labs. The animals had free access to food and water and were kept at 23°C with a 8:16 h light/dark cycle for a 4–6-week acclimatization period in our animal facility. Subsequently, in order to obtain arousal and torpor experimental groups, some animals were transferred to a special chamber that allowed the control of the temperature and photoperiod—two essential factors that affect hibernation. We designed this chamber (developed by Tiselius s.l.) with 6 individual cages to induce hibernation based on previous studies (Arendt et al., 2003). The chamber makes it possible to gradually reduce the temperature (LM35 sensors), control the illumination (adjustable LED RGB that controls intensity and color) and monitor the hamsters by measuring the general locomotor activity with a PIR (passive infrared) sensor mounted on top of each cage. Furthermore, we recorded all data obtained in a notebook computer to distinguish between the torpor and arousal phases during the hibernation cycle using the software package Fastwinter1.9 (developed by Tiselius s.l.). Hibernating animals showed torpor phases with a period of inactivity of 24 h whereas non-hibernating euthermic animals did not. The status of the animals was confirmed by body temperature measurements (infra-red thermometer) since the body temperature of a hibernating hamster falls to almost 5°C, whereas it is about 33°C in euthermic animals. As hibernation initiation proceeds with non-regular torpor bouts, we considered animals to be torpid only when they had completed three full bouts of torpor before they were sacrificed. Arousal from torpor was initiated by rising the temperature of animals artificially with a thermal blanket. Arousal animals were sacrificed 90 min later once they had been taken out of the hibernation cage. In this study, animals were compared at three stages: control or euthermic (*n* = 7), torpor (*n* = 9), and arousal (*n* = 5).

For immunocytochemical experiments, control animals and animals from different hibernation states (torpor and arousal) were sacrificed by a lethal intraperitoneal injection of sodium pentobarbital (40 mg/kg) and were then perfused intracardially with a saline solution (together with heparin) followed by 4% paraformaldehyde in 0.1 M phosphate buffer (PB, pH 7.4). The brain of each animal was removed and postfixed by immersion in the same fixative for 24 h at 4°C. Serial coronal sections (50-μm thick) were obtained with a Vibratome (St Louis, MO, USA).

### Immunofluorescence

Sections were first rinsed in PB and preincubated for 1 h at room temperature in a stock solution containing 3% normal serum of the species in which the secondary antibodies were raised (Vector Laboratories, Burlingame, CA, USA) diluted in PB with Triton X-100 (0.25%). Thereafter, the sections were incubated for 48 h at 4°C in the same stock solution containing the following primary antibodies, either alone or in the combinations indicated: mouse anti-AT8 (Pierce Endogen, 1:2000), mouse anti-GM130 (BD, 1:50), rabbit anti-MG160 (Abcam, 1:100), and rabbit anti-Golgin84 (Santa Cruz, 1:500). After rinsing in PB, the sections were incubated for 2 h at room temperature in the appropriate combinations of Alexa 488- or Alexa 594-conjugated goat anti-mouse or goat anti-rabbit antibodies (1:2000; Molecular Probes, Eugene, OR, USA). Sections were also stained with the nuclear stain DAPI (4,6 diamino-2-fenilindol; Sigma, St. Louis, MO, EEUU). Finally, the sections were washed in PB, mounted in antifade mounting medium (ProlongGold, Invitrogen) and studied by confocal microscopy (Zeiss, 710). Z sections were recorded at 0.35 μm intervals through separate channels, and ZEN 2012 software (Zeiss) was then used to construct composite images from each optical series by combining the images recorded through the different channels (image resolution: 1024 × 1024 pixels; pixel size: 0.11 μm). Colocalization of different pairs of Golgi markers was studied in double-stained sections with the aid of ZEN-lite 2012 software (Zeiss) estimating the Manders coefficient in cropped confocal stacks including complete single neurons (15 neurons per region and animal). Fiji software (3D Object counter) was used to analyze the volume and surface area of the puncta immunostained for the different GA markers in image stacks.

To determine differences between values obtained in control, torpor, and arousal groups, Kruskal-Wallis one-way analysis of variance was performed followed by Bonferroni-corrected Mann-Whitney *U*-test for pairwise comparisons, using SPSS software (version 22). To study possible differences between different regions and Golgi markers in the control group, Friedman test was performed. Wilcoxon test was used to compare mean values obtained in AT8-positive and -negative neurons. Adobe Photoshop (CS4) software was used to compose figures.

### DAB immunostaining

Free-floating sections were pretreated with 1.66% H_2_O_2_ for 30 min to quench the endogenous peroxidase activity, and then for 1 h in PB with 0.25% Triton-X and 3% normal goat serum (Vector Laboratories). The sections were then incubated overnight at 4°C with the mouse anti-AT8 antibody (Pierce Endogen, 1:2000) and the following day they were rinsed and incubated for 1 h in biotinylated goat anti-mouse IgG (1:200; Vector Laboratories). Antibody binding was detected with a Vectastain ABC immunoperoxidase kit (Vector Laboratories) and visualized with the chromogen 3,3′ diaminobenzidine tetrahydrochloride (DAB; Sigma- Aldrich, St. Louis, MO). After staining, the sections were dehydrated, cleared with xylene, and coverslips were applied.

### Western-blotting

Animals were sacrified by cervical dislocation followed by decapitation. Brains were extracted and protein samples were prepared from thick brain slices, extending from -1 to -4 with respect to Bregma (Morin and Wood, 2001). Tissue was homogenized with the aid of a potter and a syringe needle in a buffer containing 20 mM HEPES, pH 7.4; 100 mM NaCl; 50 mM NaF; 1% Triton X-100; 5 mM EDTA; 1 mM sodium orthovanadate; 5 mM Okadaic acid, 30 mM β-Glycerophosphate, 5 mM Sodium pyrophosphate tetrabasic and the complete protease inhibitor cocktail (Roche Diagnostics). Protein concentration was quantified by a Bradford assay and 30 μg of total protein were subjected to electrophoresis in 10% SDS-polyacrylamide gel and transferred to a nitrocellulose membrane (Schleinder & Schuell, Keene, NH). Membranes were incubated overnight at 4°C with the following primary antibodies: mouse anti-GM130 (BD, 1:500), rabbit anti-MG160 (Abcam, 1:250), rabbit anti-Golgin84 (SC, 1:500), or mouse anti beta-actin (Sigma, 1:5000). After washing, the membranes were incubated with the corresponding peroxidase conjugated secondary antibody (1:1000; Dako, Glostrup, Denmark) for 2 h at room temperature. The antibodies were visualized using ECL (Amersham, 1:1000; Dako, Glostrup, Denmark), densitometric analysis was carried out with Image J software and the differences between the mean values obtained in control, arousal, and torpor animal groups were compared by one way Kruskall Wallis (Dunn's *post-hoc* test) (GraphPad Prism, version 5).

## Results

### Distribution of golgi proteins in cortical neurons of euthermic hamsters

**Figure 1:**
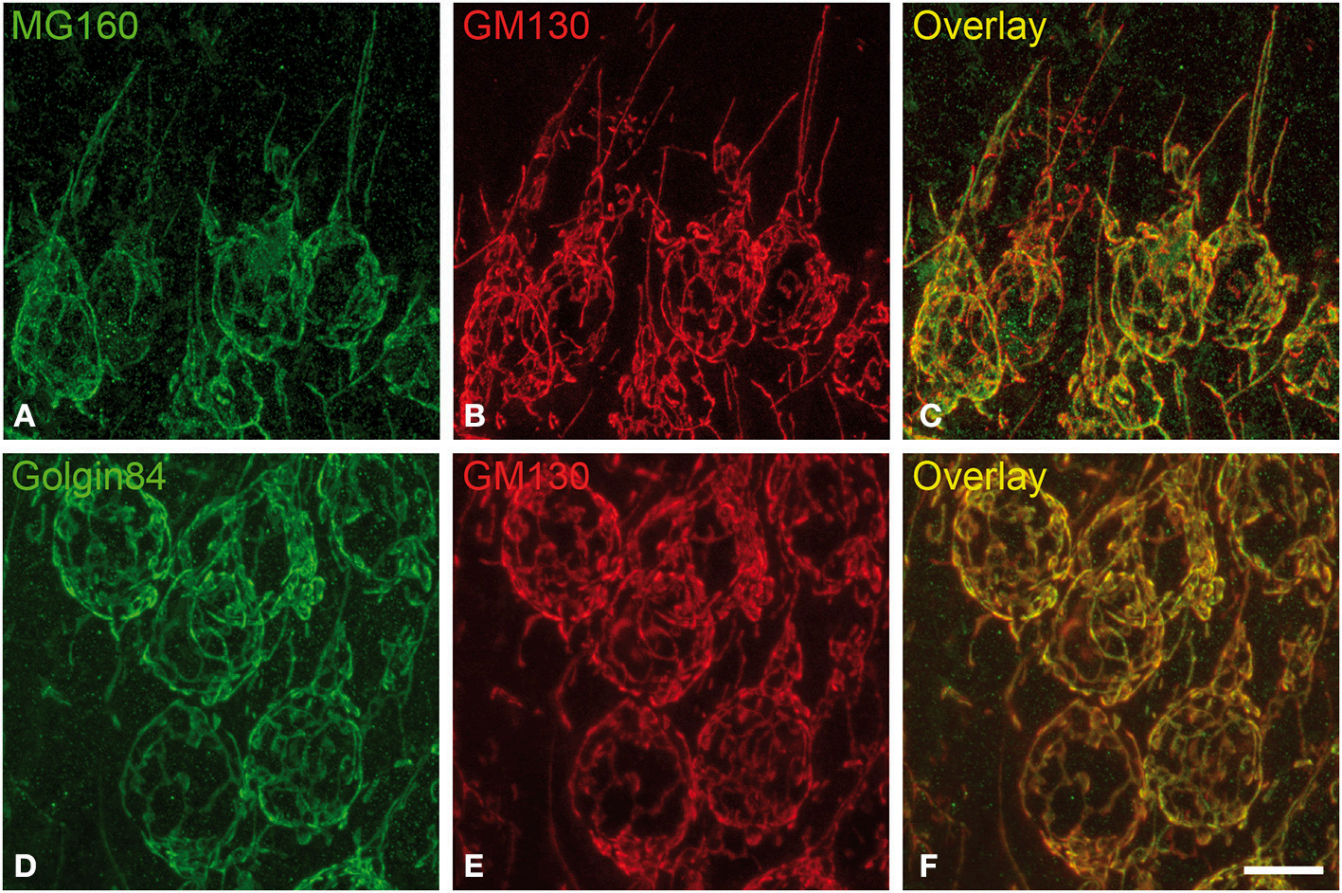
**Distribution of GA proteins in cortical neurons from euthermic hamsters. (A–F)** Pairs of images taken from hippocampal sections double-immunostained for MG160/GM130 **(A–C)** and Golgin84/GM130 **(D–F)** showing their distribution in the GA of CA1 pyramidal neurons from euthermic hamsters. Note the similar distribution patterns and the high degree of colocalization. Scale bar in **(F)** indicates 9.5 μm.

To characterize possible alterations during the hibernation cycle in the Golgi apparatus (GA) of neocortical and hippocampal neurons of Syrian hamsters, we first performed experiments with immunocytochemical staining using antibodies directed against GM130, MG160, and Golgin84 to study their distribution in euthermic hamsters (Figure 1).

It has been previously established that GM130 is mainly localized in the *cis*-Golgi compartment, MG160 is localized in the medial cisternae and golgin-84 is present throughout the Golgi stacks with an increasing gradient toward the trans-side (see Introduction for references). In spite of this, the light microscopy observations in the present study revealed a similar morphological appearance of the GA in sections immunostained for each of the three GA markers (Figure 1). The GA in hippocampal neurons consisted of a network of twisted and convoluted cisternae and tubular structures with a ribbon-like appearance, and was distributed throughout the cell body around the nucleus and partially extended to the apical dendrite (Figure 1). In neocortical neurons, the GA, as revealed with the three markers, also showed a ribbon-like appearance although it had a less complex appearance, that is, it had a lower degree of cisternae extension and convolution than in the case of hippocampal neurons (Figures S1, S2).

**Figure 2:**
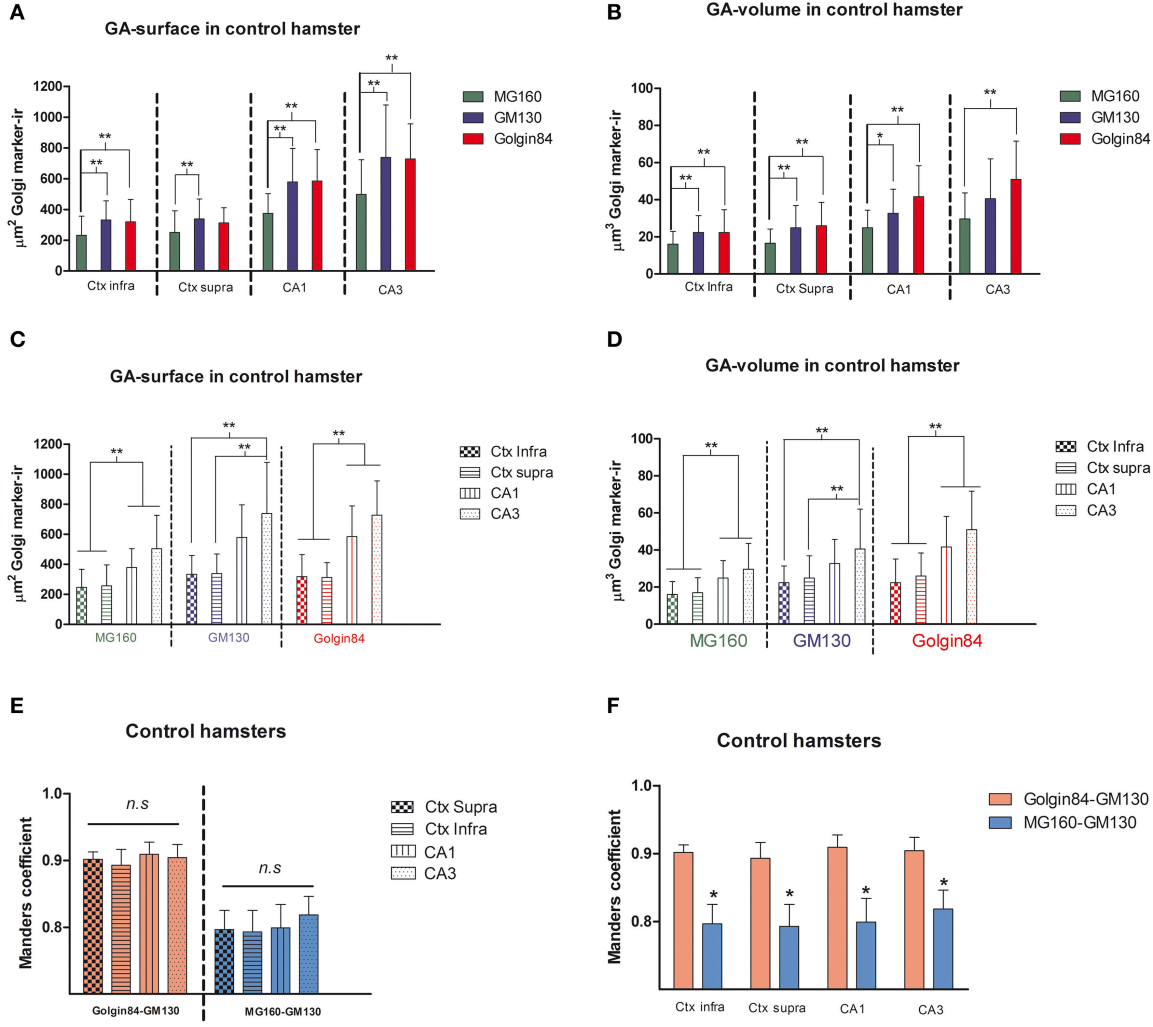
**(A–D)** Histograms showing surface area **(A,C)** and volume **(B,D)** values (mean ± SD) of GA elements immunoreactive for MG160, GM130, and Golgin84, obtained from cropped confocal image stacks (*n* > 15 in all cases) including complete single pyramidal neurons from supra and infragranular neocortical layers and CA1 and CA3 hippocampal regions. **(A,B)** Show the statistical comparisons between mean values (surface area and volume respectively) obtained with the different Golgi markers within each brain region. **(C,D)** Show the comparisons of the values obtained with each marker across the different brain regions. (Kruskall-Wallis, ^*^p ≤ 0.01; ^**^p ≤ 0.001). **(E,F)** Show comparisons across the different brain regions and within each brain region, respectively, of the mean values of Manders colocalization coefficient obtained for MG160/GM130 and Golgin84/GM130. Values for each marker combination and region were obtained from every image confocal plane of cropped image stacks corresponding to 15 fully reconstructed neurons. Kruskall-Wallis test found significant differences in the comparisons of every possible combination of mean values (*p* ≤ 0.001).

Using the 3D object counter tool in the Fiji software package, we quantified the volume and the surface area of GA elements immunoreactive for the three markers in pyramidal neurons (identified by the pyramidal shape of the soma and presence of an apical dendrite) from supra- and infra-granular layers of the neocortex, and from CA1 and CA3 hippocampal regions. The results indicate that in all regions analyzed MG160 shows, in general, a more restricted distribution in the GA than Golgin84 and GM130 (Figures 2A,B). In addition, when values from the different neuronal populations were compared, the GA in hippocampal neurons, especially in CA3 neurons, stained with the three different markers, showed a significantly larger surface area and volume than in neocortical pyramidal neurons from supragranular and infragranular layers (Figures 2C,D).

To gain a deeper insight into the distribution patterns of the different GA markers in euthermic hamsters, we then quantified their degree of colocalization in pyramidal neurons from neocortex and CA1 and CA3 hippocampal regions in MG160/GM130 and GM130-Golgin84 double-immunostained sections (Figures 1, 2E,F). In all neuronal populations analyzed, a lower Manders coefficient of colocalization (Manders et al., 1993) was found for MG160/GM130 than for GM130/Golgin84 (with “0” representing a lack of colocalization and “1” representing complete colocalization). This was probably due to the wide distribution of GM130 in the GA and the more restricted distribution of MG160. Despite the similar morphological appearance of the GA, revealed by immunostaining with the three different antibodies in the present study, the lack of complete colocalization in the MG160/GM130 and GM130/Golgin84 double-stained sections indicate the existence of microdomains in the GA where the two proteins tested in each combination are not co-expressed (Figures 1C,F).

### Differential effects of hibernation on the expression of golgi proteins

**Figure 3:**
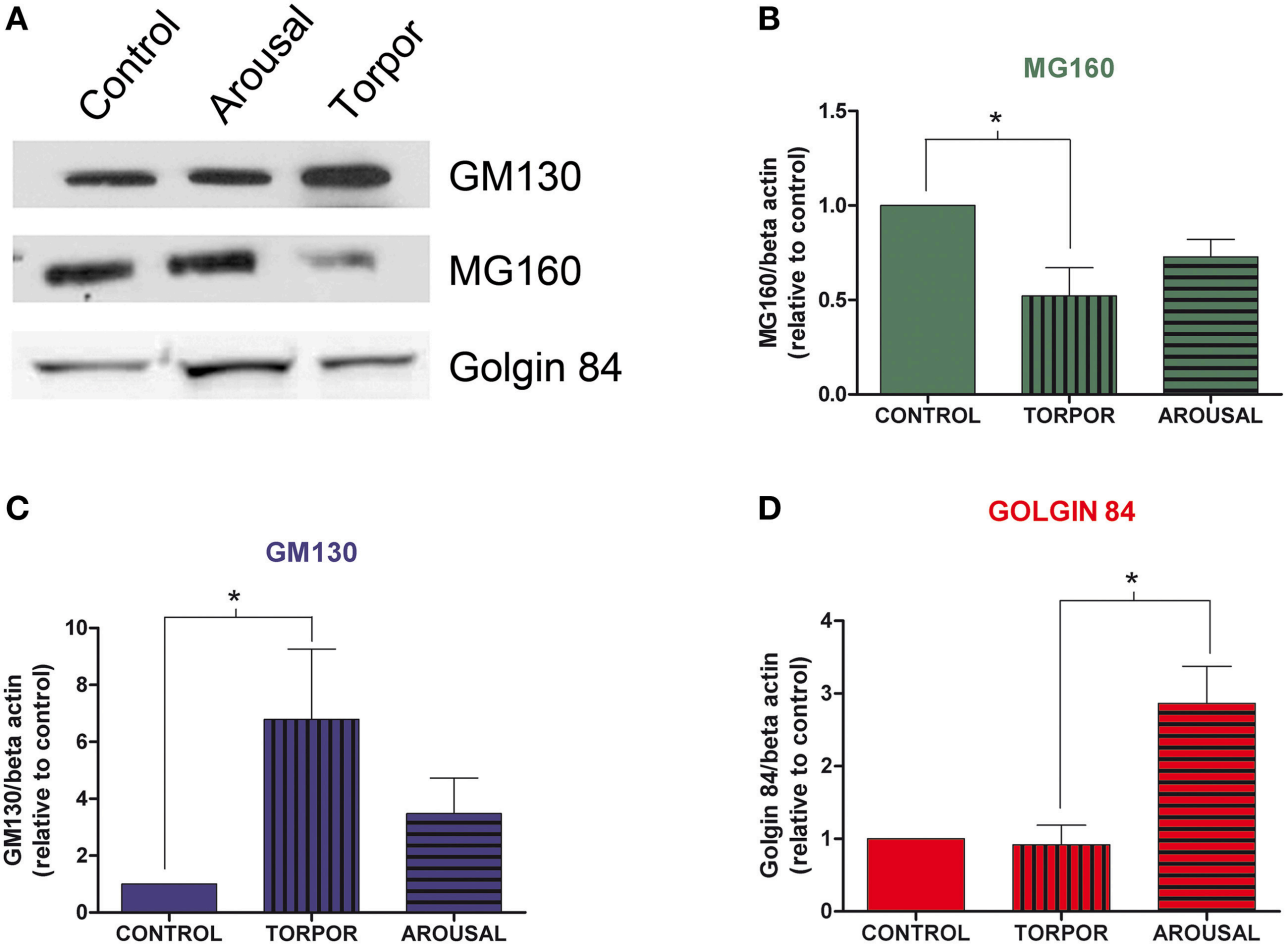
**Alterations in Golgi protein expression during the hibernation of Syrian hamsters. (A)** Western blot of GM130, MG160, and Golgin84 in protein samples extracted from euthermic (control) and hibernating (arousal and torpor phase) hamsters. Histograms show GM160 **(B)**, MG130 **(C)**, and Golgin84 **(D)** expression normalized to β-actin. The data represent the mean ± SD of three independent experiments. Note the increase of GM130 and the decrease of MG160 expression in arousal and more markedly in torpor as compared to control animals. Also note the high Golgin84 expression levels during arousal (Kruskall-Wallis, ^*^p ≤ 0.05).

We then analyzed the possible variations of Golgin84, MG160, and GM130 expression during the hibernation cycle by examining Western blots of protein samples obtained from animals sacrificed in the different hibernation stages (Figure 3). The analysis revealed that torpor bouts differentially affected expression levels of Golgi markers normalized to the expression of b-actin (Figure 3). Levels of MG160 decreased in samples from animals in torpor state compared to control expression levels, whereas expression of Golgin84 remained unaltered and GM130 expression increased (Figure 3). During arousal, MG160 and GM130 showed intermediate levels of expression, that is, between control and torpor levels. By contrast, in two cases, the expression of Golgin84 was more intense in samples from arousal animals than during euthermia or torpor states (Figure 3).

**Figure 4:**
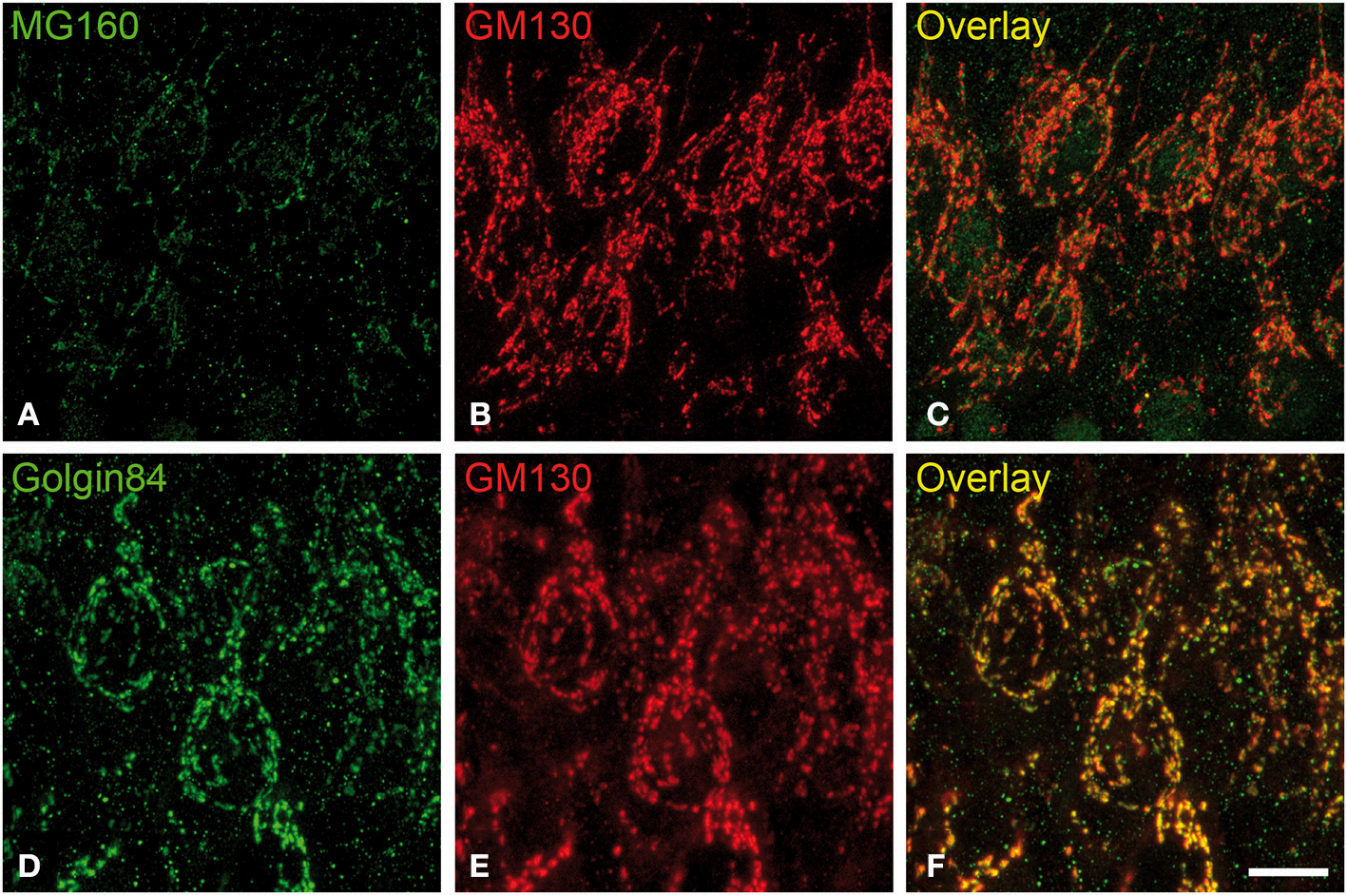
**Distribution of GA proteins in cortical neurons from torpid hamsters. (A–F)** Pairs of images taken from hippocampal sections double-immunostained for MG160/GM130 **(A–C)** and Golgin84/GM130 **(D–F)** showing their distribution in the GA of CA1 pyramidal neurons from hamsters at torpor. Note the reduction of MG160 immunostaining and the strong fragmentation of the GA as revealed with the different Golgi markers. Scale bar in **(F)** indicates 9.5 μm.

**Figure 5:**
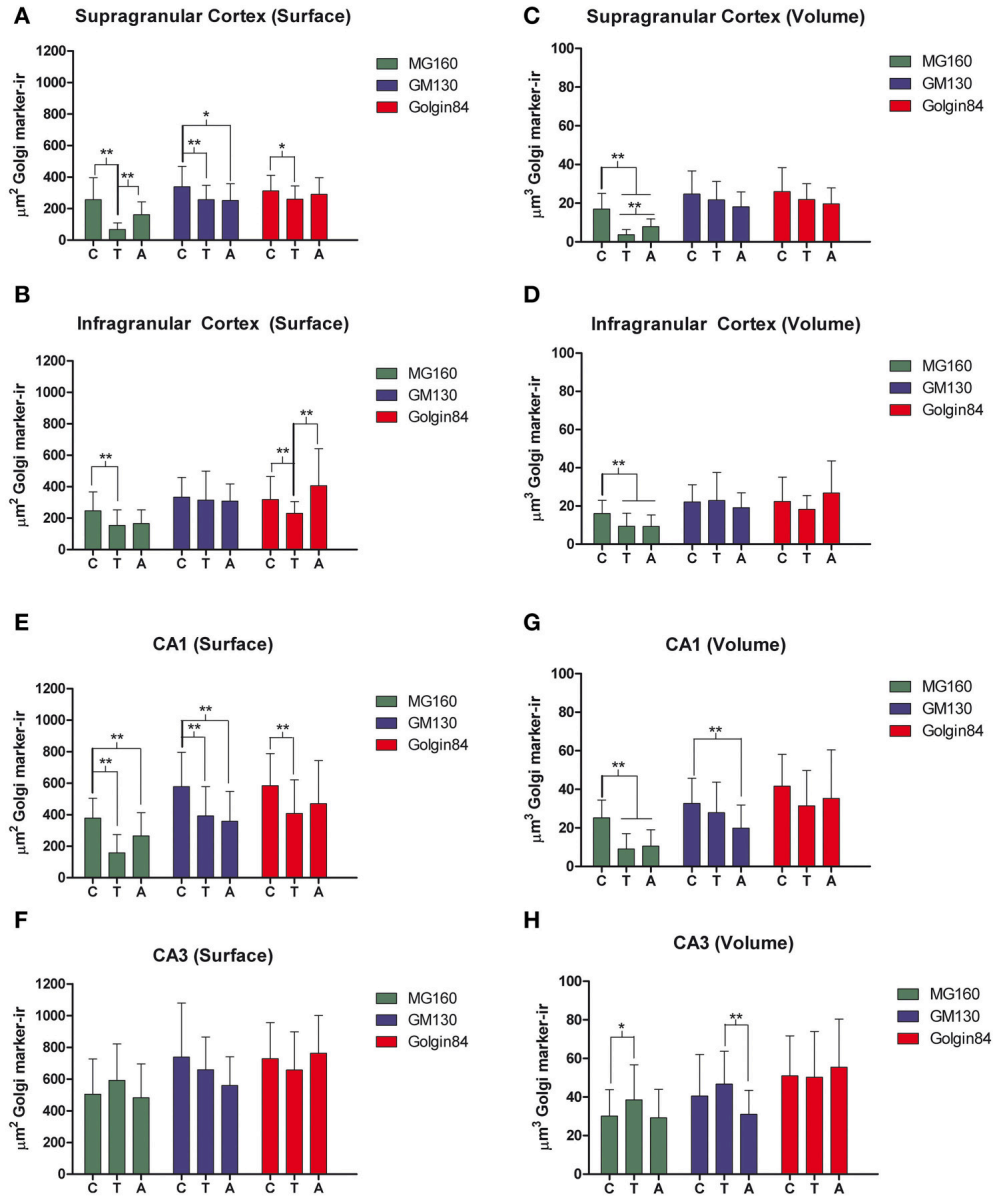
**Histograms showing volume and surface area values (mean ± SD) of GA elements immunoreactive for MG160, GM130, and Golgin84, obtained from cropped confocal image stacks (***n*** > 15 in all cases) including complete single pyramidal neurons from supra (A,C) and infragranular (B,D) neocortical layers and CA1 (E,G) and CA3 (F,H) hippocampal regions from euthermic (control) animals and animals during arousal and torpor**. Kruskall-Wallis, ^*^p ≤ 0.01; ^**^p ≤ 0.002; c, control; t, torpor; a, arousal.

**Figure 6:**
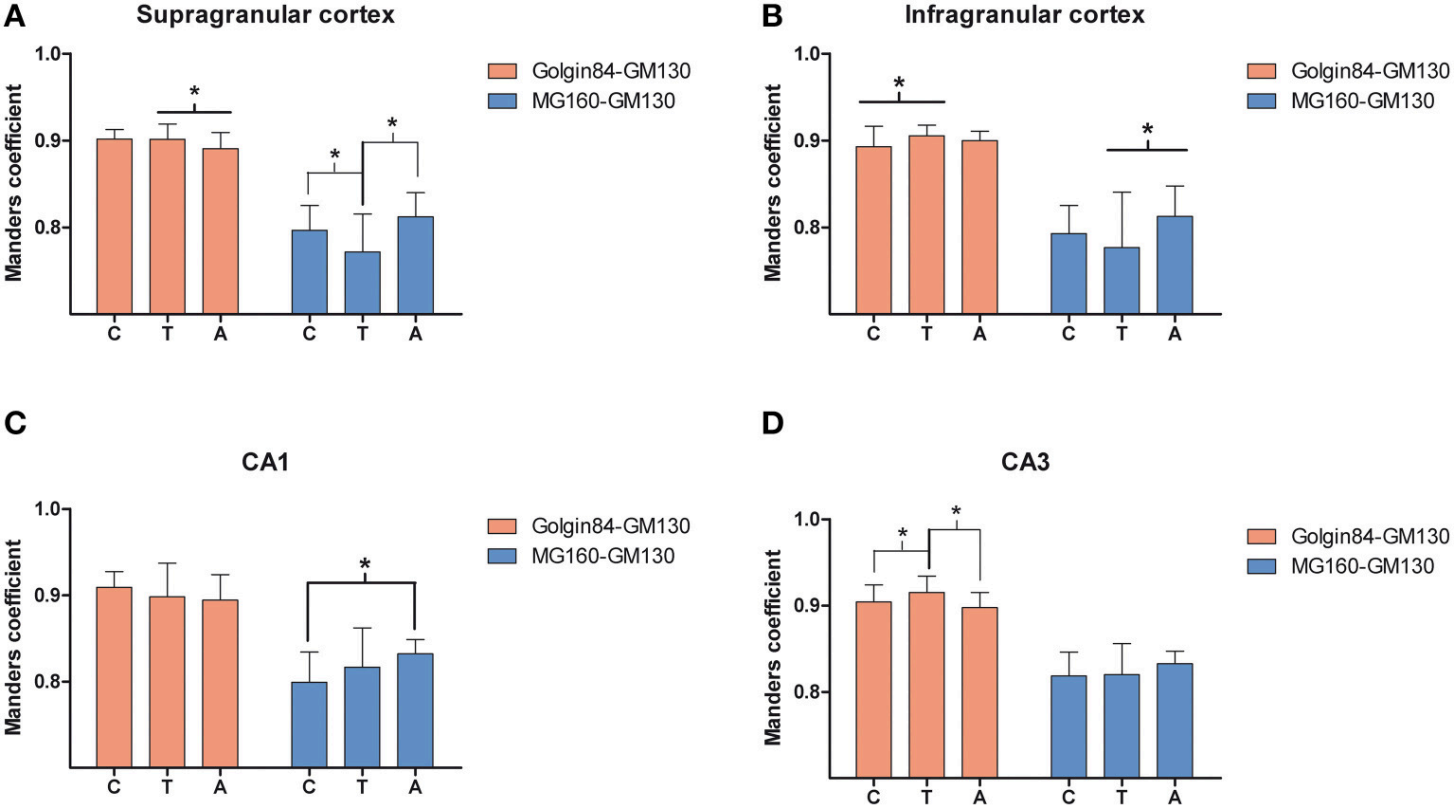
**Comparisons in supra (A) and infragranular (B) neocortical layers and in the CA1 (C) and CA3 (D) region of the hippocampus between mean values of Manders colocalization coefficient obtained for MG160/GM130 and Golgin84/GM130 in control, arousal and torpor animals**. Values for each marker combination, region and animal were obtained from every image confocal plane of cropped image stacks corresponding to 15 fully reconstructed neurons. Kruskall-wallis test found significant differences in the comparisons of every possible combination of mean values (^*^p ≤ 0.05). c, control; t, torpor; a, arousal.

We then analyzed, in immunocytochemically stained material, whether the hibernation-related changes in protein expression levels observed by Western blot were reflected in alterations in intensity or distribution patterns of Golgi markers in neocortical and hippocampal neurons (Figures 4–6, Figures S1, S2). As the levels of MG160 expression decreased in arousal and especially in torpor, observed by Western Blot, there was a corresponding reduction in the intensity of MG160 immunostaining (Figure 4, Figure S1). This reduction was more evident in neocortical neurons and CA1 pyramidal cells than in CA3 pyramidal neurons in which MG160 immunostaining was well preserved although MG160-ir elements did display a fragmented appearance (Figure 4, Figure S1). Alterations in MG160 expression were evidenced by a significant reduction in the surface area and volume of immunoreactive elements of MG160-ir elements in all neuronal populations analyzed, except CA3 pyramidal neurons, in which these parameters were slightly increased in torpid animals (Figure 5). Accordingly, in somatosensory cortex, but not in the hippocampus, decreases in the MG160/GM130 Manders colocalization coefficient were found in torpor as compared to control and arousal states (Figure 6).

No changes were found in the apparent intensity of GM130 immunostaining in hibernating hamsters. However, profound morphological modifications of GM130-ir elements of the GA were found; in all regions analyzed, they showed a fragmented appearance in arousal and particularly in torpor animals as compared with controls (Figures S1, S2). This fragmentation was accompanied by a decrease in the volume and surface area of GM130-ir elements during hibernation, which reached statistical significance especially in the CA1 region of the hippocampus (Figure 5). Similar alterations to those found with GM130 were observed for Golgin84 immunostaining in torpid animals. These included—without apparent changes in intensity of Golgin84 immunostaining—an intense fragmentation (Figure 4, Figures S1, S2) and a decrease in surface area, but not volume, of Golgin84-ir elements, which reached statistical significance in infragranular neocortical and CA1 neurons (Figure 5). These changes were accompanied by an increase in the GM130/Golgin84 colocalization Manders coefficient during torpor in all areas except in CA1 (Figure 6).

### Golgi apparatus changes in neurons with hyperphosphorylated tau during torpor

**Figure 7:**
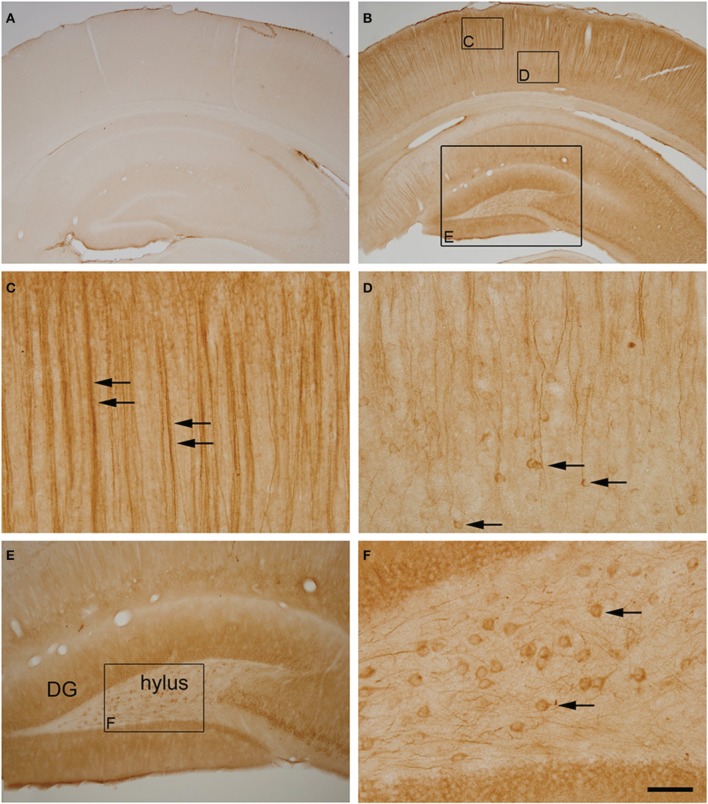
**(A,B)** Photomicrographs showing the patterns of hyperphosphorylated tau immunostaining (AT8 antibody) in the neocortex and hippocampus of control **(A)** and torpid **(B)** Syrian hamsters. Small squared zones in **(B)** are shown at higher magnification in **(C,D)**. Note the AT8 immunostaining of layer V neuronal cell bodies (arrows in **D**) and their apical dendrites in layer III (arrows in **C**). The large squared zone in **(B)** and squared zone in **(E)** are shown at higher magnification in **(E,F)**, respectively. Note the intense AT8 immunostaining in hylar neurons (arrows in **F**). Scale bar in F indicates 570 μm in **(A,B)**, 57 μm in **(C,D,F)** and 115 μm in **(E)**.

**Figure 8:**
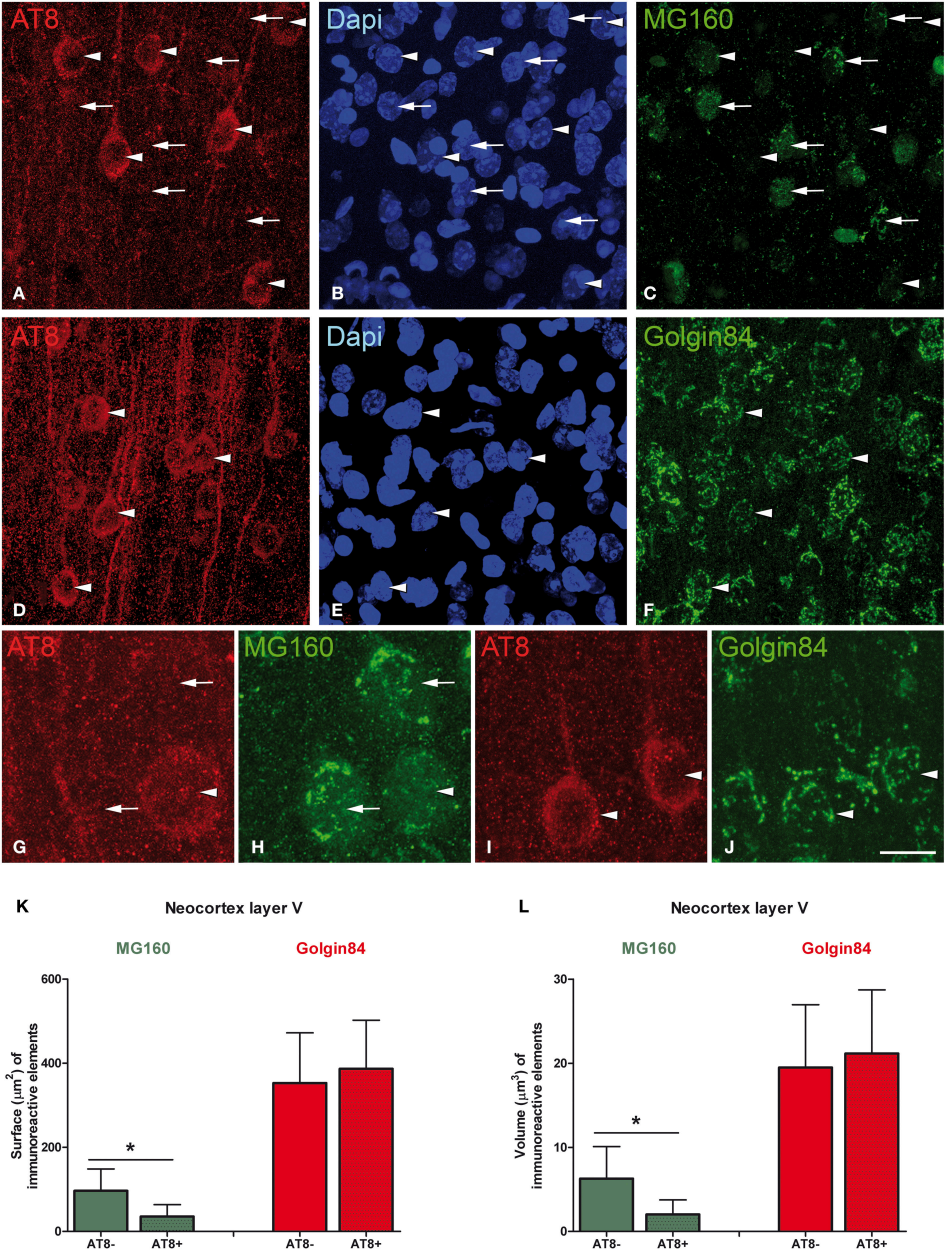
**Microphotographs taken from layer V of the somatosensory cortex from Syrian hamsters in torpor state**. Sections were double stained with antibodies directed against AT8 and MG160 **(A–C,G,H)**, and against AT8 and Golgin84 **(D–F,I,J)**, counterstained with Dapi. Arrowheads indicate AT8 positive neurons whereas arrows indicate adjacent AT8 negative neurons. Note the marked reduction in MG160 immunostaining of the GA in AT8+ neurons and the similar pattern of Golgin84 immunostaining between AT8+ and AT8− neurons. Histograms show surface area **(K)** and volume **(L)** values (mean ± SD) of GA elements immunoreactive for MG160 and Golgin84 in AT8+ cells and in AT8− cells. Wilcoxon test found significant differences in the comparisons between AT8+ and AT8− cells in both volume and surface area values of MG160-ir (^*^p ≤ 0.001) but not Golgin84-ir elements. Scale bar in **(J)** indicates 20 μm in **(A–F)**, 7 μm in **(G,H)** and 10.5 μm in **(I,J)**.

Previous studies have described that during the torpor phase of hibernation tau protein is highly phosphorylated in cortical neurons, whereas after arousal this high phosphorylation decreases to normal levels (Arendt et al., 2003; Härtig et al., 2005, 2007; León-Espinosa et al., 2013). In accordance with these studies, the present results (from brain sections immunocytochemically stained with AT8 antibody which recognizes phospho Ser202 and phospho Thr205 sites in Tau protein) indicate that, in the Syrian hamster, tau hyperphosphorylation is particularly evident in a subpopulation of pyramidal neurons in layer V of the neocortex and in neurons of the CA3 and hilar regions of the hippocampus (Figure 7). Next we studied whether neurons with hyperphosphorylated tau were more or less prone to undergo morphological alterations in the GA during the hibernation cycle. For this purpose, in the somatosensory cortex of hamsters at torpor, we analyzed confocal image stacks from layer V neurons double immunostained for AT8 and MG160 to examine whether alterations in MG160 expression in the GA were more pronounced in neurons with tau hyperphosphorylation (AT8-positive) than in AT8-negative neurons. We found that the mean volume and surface area of MG160-ir elements were significantly lower in AT8+ neurons than in the surrounding AT8− neurons, although not all AT8+ neurons were equally affected (Figure 8). However, this does not seem to be the case in the hippocampus, where the reduction in GA MG160 immunostaining was more pronounced in CA1 than in CA3 neurons (Figure 5) even though AT8 immunostaining was more intense in CA3 than in CA1 (Figure 7).

Finally, in AT8/Golgin84 double-immunostained sections from torpor animals, we observed that the surface area and volume of Golgin84-ir GA elements were similar in AT8+ neurons and AT8- neurons (Figure 8). These results indicate that the accumulation of hyperphosphorylated tau at Ser202 and Thr205 during the torpor stage of hibernation seems to be independent of the concomitant reversible shrinkage and fragmentation of the GA.

## Discussion

**Figure 9:**
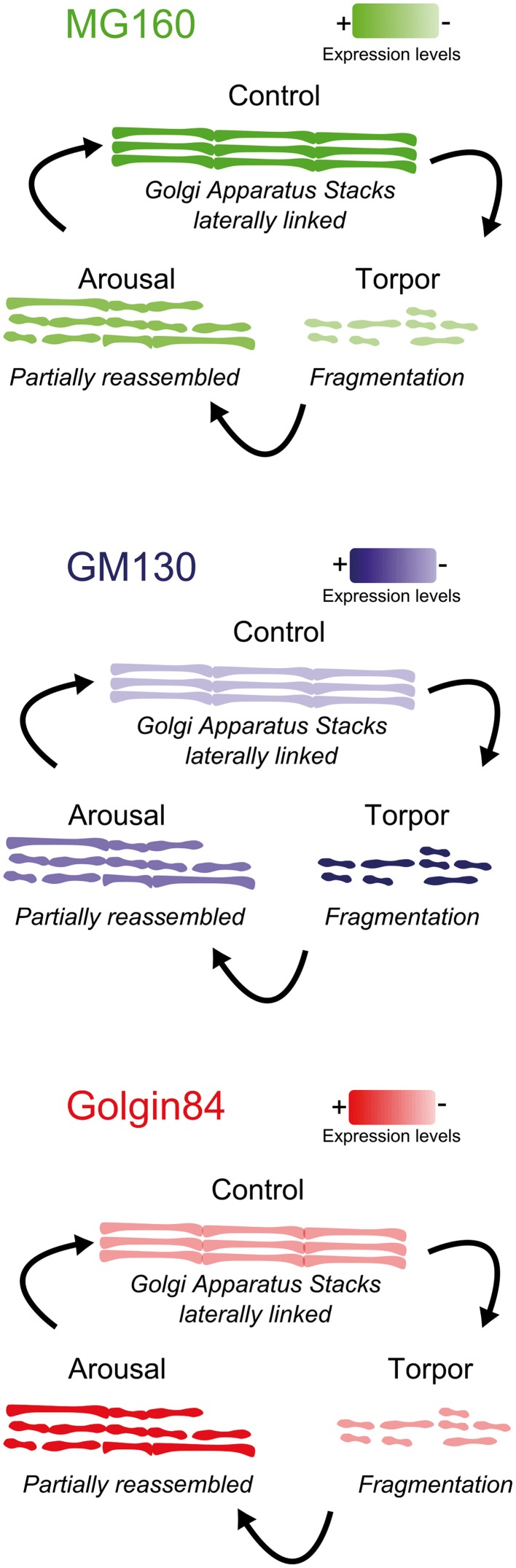
**Summarizing diagram showing the structural alterations of the GA during the hibernation cycle along with changes in the expression levels of the GM130, MG160, and Golgin84 GA proteins**.

The main results of the present study, schematically summarized in Figure 9, show that MG160 and, to a wider extent, GM130 and Golgin84 are expressed in the GA of neocortical and hippocampal neurons in euthermic Syrian hamsters. We also showed quantitatively that during the hibernation cycle, the GA undergoes significant and protein-specific changes in expression levels and distribution patterns of these Golgi protein components. Furthermore, these changes were accompanied by a transitory Golgi fragmentation with reductions in the volume and surface area of the GA during torpor and arousal stages that varied between different regions. We also found selective changes in the GA in neurons expressing high levels of hyperphosphorylated tau.

### Fragmentation of the golgi apparatus during hibernation

The GA in nerve tissue becomes fragmented in a variety of pathological conditions as already noted since the times of Cajal in cells at the edges of mechanical lesions (DeFelipe and Jones, 1991). Later numerous studies have reported Golgi fragmentation in apoptotic cells (Aslan and Thomas, 2009) and in a variety of pathological conditions including Alzheimer's disease, amyotrophic lateral sclerosis, Creutzfeldt-Jacob disease, multiple system atrophy, Parkinson's disease, spinocerebellar ataxia type 2 and Niemann-Pick type C (Gonatas et al., 1992; Dal Canto, 1996; Stieber et al., 1996; Sakurai et al., 2000, 2002; Walkley and Suzuki, 2004; Fujita et al., 2006; Eschbach and Dupuis, 2011). It is well known that GA in different cell types undergoes a transitory fragmentation during mitotic cell division along with some of the mechanisms that regulate this fragmentation. During mitosis, the GA is disassembled in early prophase with a splitting of GA into small, round, disconnected and dispersed elements, and is reassembled in telophase (Levine et al., 1995; Corda et al., 2012). Previous studies have demonstrated the involvement of the Golgi protein tether complex, including GM130, GRASP65, p115, giantin, and Rab GTPases, in the stacking and lateral tethering of Golgi cisternae that allow ribbon formation and the regulation of the Golgi disassembly and reassembly during mitosis (Nakamura et al., 1995; Barr et al., 1997, 1998; Shorter and Warren, 1999; Puthenveedu and Linstedt, 2001, 2005; Puthenveedu et al., 2006; Nakamura, 2010). Along with the tether complex, Golgin84 was also suggested to play a key role in the assembly and maintenance of Golgi ribbon in mammalian cells (Diao et al., 2003). The cisternal unstacking and the disassembly of the GA at the onset of mitosis have been related to the inhibition of p115 binding to GM130 by GM130 phosphorylation and to the phosphorylation of Grasp65 (Nakamura et al., 1997; Lowe et al., 1998, 2000; Puthenveedu and Linstedt, 2001; Wang et al., 2003, 2005; Nakamura, 2010).

In the present study, we demonstrate that during hibernation, the GA of hippocampal and neocortical neurons undergoes a pronounced morphological reorganization in Syrian hamsters. A strong fragmentation along with a volume decrease of the GA in torpid animals was found by immunostaining for GM130, MG160, and Golgin84. Future electron microscopic observations would be necessary to determine the extent of the Golgi ribbon fragmentation during hibernation and establish whether it affects the integrity of the Golgi stacks. However, previous electron microscopy observations in taste bud cells of hibernating ground squirrels noted a transitory fragmentation or disassembly of the GA during the torpor phase in which dictyosomes were rarely seen and instead clusters of small vesicles appeared (Popov et al., 1999; Bocharova et al., 2011). Although, the mechanisms underlying neuronal Golgi fragmentation during torpor remain to be explored, the present Western Blot observations revealed a drop in whole brain MG160 brain expression levels along with an increase in those for GM130 in torpid animals as compared with euthermic animals. This was accompanied by an important reduction in the levels of MG160 immunostaining of the GA in hippocampal and neocortical neurons. The down-regulation of MG160 expression during torpor could participate in the mechanisms involved in the fragmentation of the GA since MG160 has been proposed to play important roles in the biogenesis and functioning of the GA (Gonatas et al., 1995, 1998a).

The present Western blot data also showed that brain expression levels of Golgin84 are transiently increased during arousal. The interference with Golgin84 expression has been shown to disrupt the Golgi ribbon and induce the appearance of Golgi fragments in HEK293/tau cells (Jiang et al., 2014). In addition, overexpressing of Golgin84 rescued brefelding-A-induced Golgi alterations, indicating the important role of Golgin84 in the structural maintenance of the GA (Jiang et al., 2014). Therefore, it is tempting to speculate that the up-regulation of Golgin84 expression during arousal (present results) is required for the rapid rebuilding or reorganization of the Golgi ribbon apparatus during arousal leading the animals from torpor to euthermia. Although, the biological relevance of these arousals is still not known, previous reports indicate that they could be related to a neuroprotective role (Daan et al., 1991; Arendt et al., 2003). Our results may suggest that a rapid GA structure recovery takes place during arousal, as a GA reorganization is needed for the adaptation of neurons to arousal-torpor bouts.

Hibernation is a state of low energy availability (Humphries et al., 2003) with a virtual cessation of neuronal activity in cortical and midbrain areas (Strumwasser, 1959; South, 1972; Walker et al., 1977; Igelmund, 1996). Given that the size of the GA has been directly related to the level of cell activity (Lucassen et al., 1993; Salehi et al., 1994), the fragmentation and reduction of the GA observed in the present study is probably related to a reduced neuronal capacity for protein processing, modification and targeting during torpor. This is in line with the hibernation-related reductions in cell body area, dendritic arbor complexity and spine density previously reported (Popov et al., 1992; Popov and Bocharova, 1992; von der Ohe et al., 2006) and the general decrease in energetically expensive processes such as transcription and protein synthesis (Strumwasser, 1959; Walker et al., 1977; Zhegunov, 1988; Rolfe and Brown, 1997; Frerichs et al., 1998; Popov et al., 1999; Buck and Barnes, 2000; van Breukelen and Martin, 2001, 2002). However, changes in protein expression and localization during hibernation seem to be complex, highly regulated, protein-specific processes. For instance, brain expression patterns of the three GA proteins evaluated in the present study by Western Blot and immunocytochemistry are differentially regulated during torpor and arousal in Syrian hamsters. In addition, in hibernating ground squirrels a rapid and transitory dissociation of pre- and post-synaptic proteins from synapses have been reported during torpor in parallel with dendritic retractions and loss of synaptic connectivity (von der Ohe et al., 2006, 2007). Whether, these processes are related to the alterations of the GA found in the present study warrants further research.

### Golgi apparatus fragmentation and tau hyperphosphorylation

It is known that the structure of the Golgi complex also depends on the integrity of the cytoskeleton. For instance, actin depolymerization causes Golgi stacks to fragment and results in swelling of cisternae (Egea et al., 2006). The disruption of the microtubules or the dynein/dynactin complex, that bind the Golgi complex to the microtubules, also causes marked changes in Golgi shape and localization (Cole et al., 1996; Burkhardt et al., 1997). It has been proposed that, among other functions, the microtubule interacting protein tau—which is localized on GA membranes—could serve as a link between the Golgi membranes and microtubules, along with many of the tau phosphorylation-related kinases which are also located in the GA (Farah et al., 2006). Therefore, the perturbation of the microtubule network by tau hyperphosphorylation could alter GA structure and the secretory pathway, and impair the highly regulated processes of protein sorting in neuronal compartments. Different studies have shown that, during the mitotic division of different cell types, GA fragmentation is concomitant with a general phosphorylation of tau which is recognized by phosphorylation-dependent antibodies used to label neurofibrillary tangles in Alzheimer's disease (AD), such as PHF1, AT8, AT100, or AT180 (Pope et al., 1994; Preuss et al., 1995; Vincent et al., 1996; Illenberger et al., 1998; Preuss and Mandelkow, 1998; Delobel et al., 2002). In AD patients, the GA of cortical neurons undergo widespread structural alterations before the formation of neurofibrillary tangles (Stieber et al., 1996). In animal models of AD, the overexpression of tau induces Golgi fragmentation in neurons leading to the suggestion that in AD brains the tau pathology precedes Golgi fragmentation (Lin et al., 2003; Liazoghli et al., 2005). However, studies in HEK293/tau cells showed that Golgi-disturbing agents, brefeldin A and nocodazole, induced tau hyperphosphorylation suggesting that Golgi fragmentation could be an upstream event triggering tau hyperphosphorylation through activation of kinases (Jiang et al., 2014). Previous studies have proposed that, in terms of tau phosphorylation, hibernating animals show some similarities to AD patients at the early stages of the disease, as neurons from torpid animals show a marked increase of hyperphosphorylated tau in contrast to euthermic animals (Zhou et al., 2001; Arendt et al., 2003; Arendt, 2004; Avila et al., 2004; Härtig et al., 2005, 2007; Su et al., 2008; Stieler et al., 2011). Our results shows that, in torpor state, the reduction in the volume and surface area of the MG160-ir elements of the GA in layer V neocortical neurons is more pronounced in AT8+ than in AT8− neurons, suggesting that hyperphosphorylated tau has a significant deleterious effect on the MG160 expression at the GA. Alternatively, the decrease in MG160 expression during torpor might have an effect on tau hyperphosphorylation at Ser202/Thr205 sites. This is supported by the fact that MG160 can bind to different FGFs, including FGF-2, reducing FGF internalization and inhibiting the intracellular accumulation of this growth factor (Burrus et al., 1992; Zhou et al., 1997; Zuber et al., 1997; Yamaguchi et al., 2003). FGF-2 levels are increased in AD (Stopa et al., 1990; Cummings et al., 1993) and it has been shown, in neural progenitor cells and in PC12 cells, that FGF-2 upregulation can increase GSK3 activity and induce the hyperphosphorylation of tau protein in various epitopes including S202, recognized by AT8 (Tatebayashi et al., 1999, 2003). Therefore, it is possible that the reduction in the MG160 expression in neocortical cells during torpor results in increased intracellular FGF levels that lead to the tau hyperphosphorylation observed with AT8 antibody. Further studies are needed to test this hypothesis both in hibernating animals, in tissue from AD patients and in cellular models of the disease.

Finally, we have shown in the present study that, during torpor, there are no differences between AT8+ and AT8− layer V pyramidal neurons immunostained with Golgin84 in terms of the reduction in volume and surface area of the GA. This indicated that not all GA proteins are affected by hyperphosphorylated tau accumulation. However, it is known from Western Blot studies that tau is hyperphosphorylated at different residues besides AT8 recognition sites during hibernation (Stieler et al., 2011). Therefore, further studies should explore whether the extent of GA alterations is correlated with degree of accumulation of tau hyperphosphorylated at sites other than Ser202/Thr205.

## Author contributions

AA and GL equal contribution. AA, GL, and AM designed research; AA and GL performed research and analyzed data; JD and AM wrote the paper.

### Conflict of interest statement

The authors declare that the research was conducted in the absence of any commercial or financial relationships that could be construed as a potential conflict of interest. The handling editor Agustin González declares that, despite being affiliated to the same university as the author Alberto Muñoz, the review process was handled objectively and no conflict of interest exists.
