# Supplementary figures and images for: Changes in the Golgi Apparatus of Neocortical and Hippocampal Neurons in the Hibernating Hamster
Source: Front Neuroanat. 2015 Dec 15;9:157. doi: 10.3389/fnana.2015.00157 (PMC4678224; doi:10.3389/fnana.2015.00157)

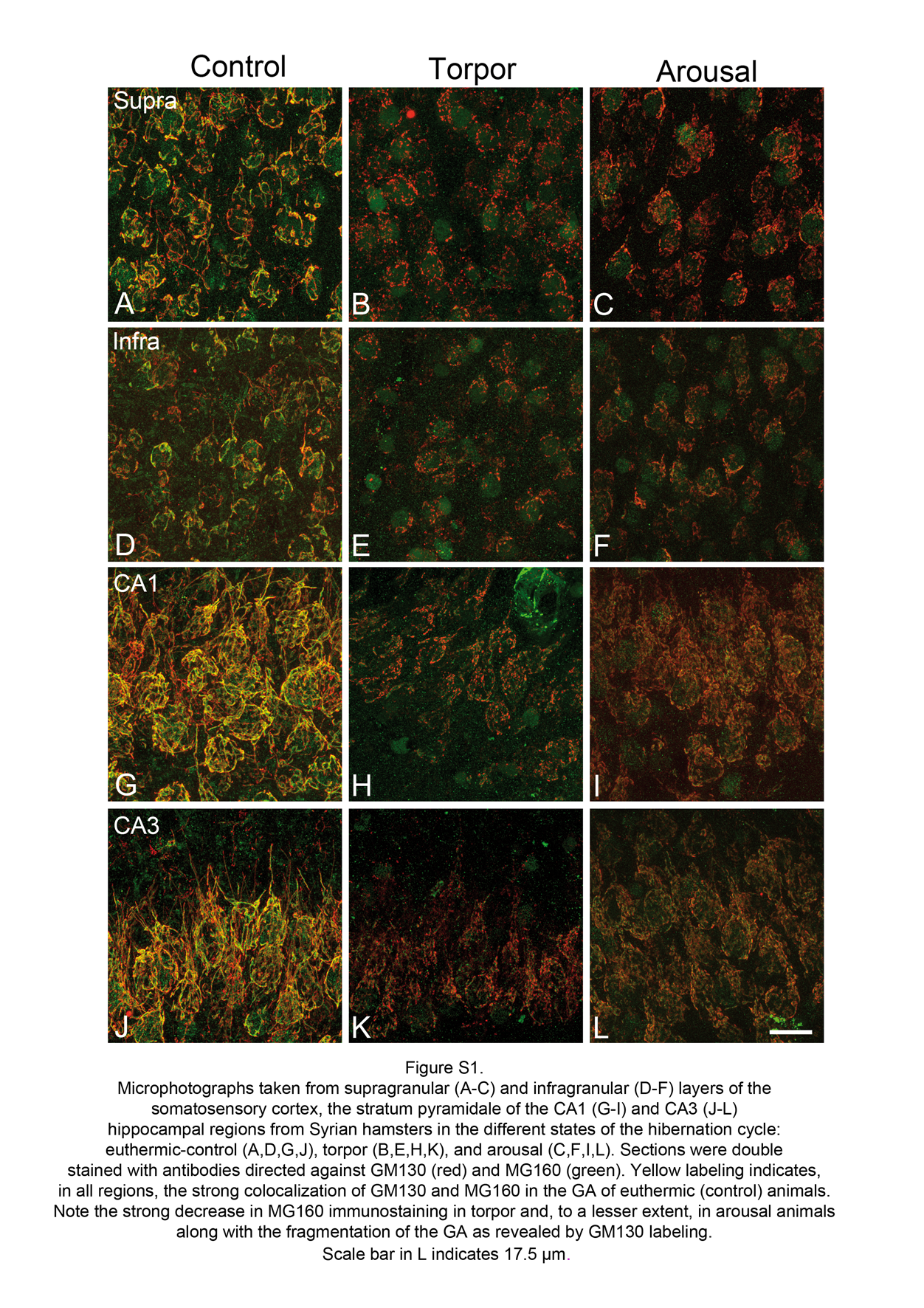

Supplement: Supplementary file 1 [file Image1.TIF]

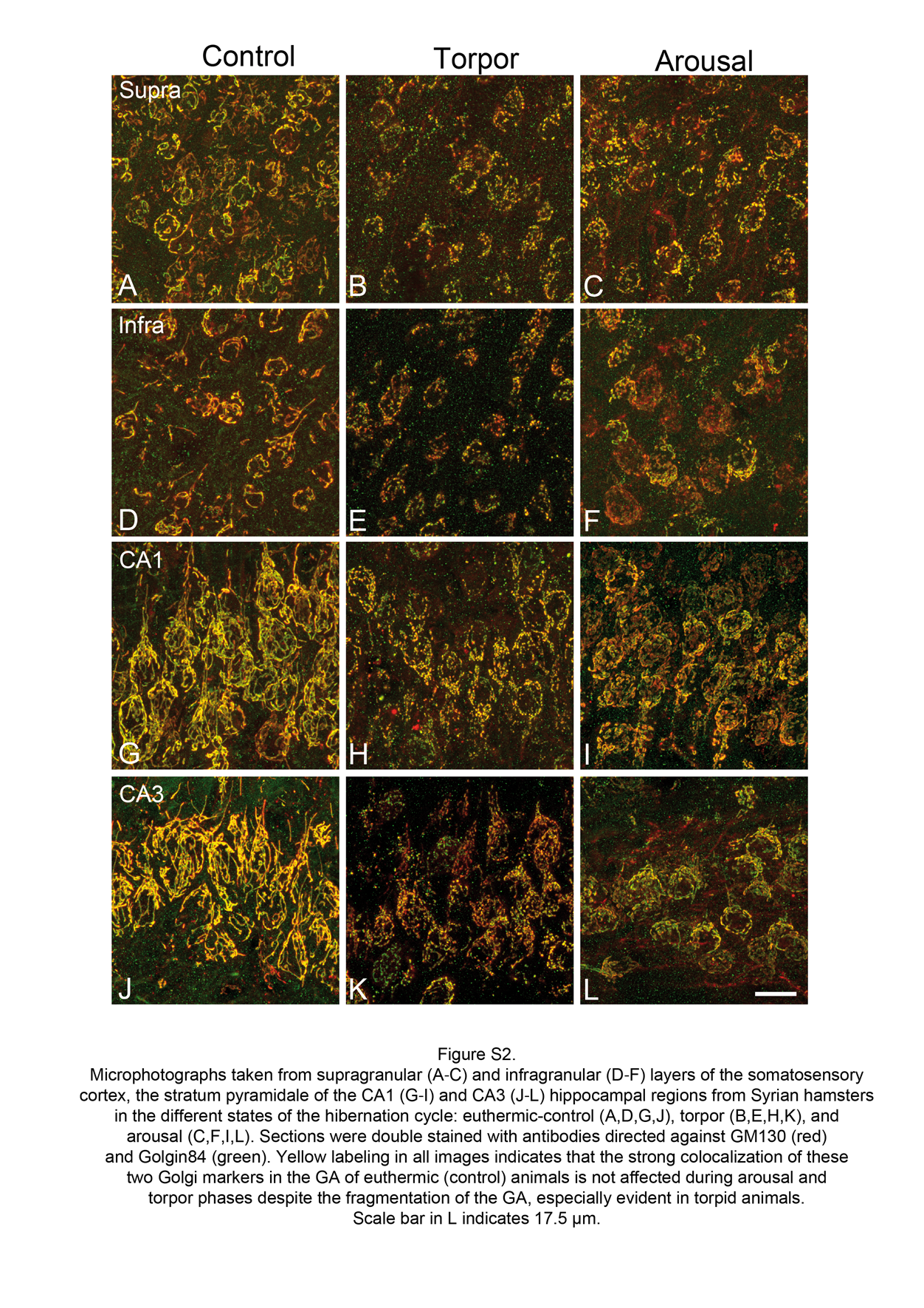

Supplement: Supplementary file 2 [file Image2.TIF]
